# The impact of the COVID-19 pandemic on TB notifications in Ukraine in 2020

**DOI:** 10.5588/ijtldopen.24.0194

**Published:** 2024-06-01

**Authors:** A.N. Shapiro, M. Dolynska, S.S. Chiang, N. Rybak, V. Petrenko, C.R. Horsburgh, J. Kobe, I. Terleieva, O. Sakalska, H.E. Jenkins

**Affiliations:** ^1^ Department of Biostatistics, Boston University School of Public Health, Boston, MA, USA;; ^2^ NGO Infection Control Ukraine, Kyiv, Ukraine;; ^3^ Department of Pediatrics, Warren Alpert Medical School of Brown University, Providence, RI, USA;; ^4^ Center for International Health Research, Rhode Island Hospital, Providence, RI, USA;; ^5^ Department of Medicine, Warren Alpert Medical School of Brown University, Providence, RI, USA;; ^6^ Bogomolets National Medical University, Kyiv, Ukraine;; ^7^ Departments of Global Health, Epidemiology, Biostatistics and Medicine, Boston University, Boston, MA, USA;; ^8^ Public Health Center of the Ministry of Health, Kyiv City, Ukraine

**Keywords:** epidemiology, modelling, drug resistance, tuberculosis, prevention, control program

## Abstract

**BACKGROUND:**

We assessed the impact of the COVID-19 pandemic on TB notifications in Ukraine, stratified by multiple subgroups.

**DESIGN/METHODS:**

We analyzed data from Ukraine’s National TB Program from January 2015 to December 2020 using interrupted time series models. We compared observed cases to counterfactual estimated cases had the pandemic not occurred and estimated trends through December 2020 nationally and by various demographics. We compared the proportions of individuals who underwent drug susceptibility testing (DST) in February 2020 and April 2020 to assess the pandemic impact on drug resistance testing.

**RESULTS:**

In April 2020, there were 39% (95% CI 36–42) fewer TB notifications than the estimated counterfactual (3,060 estimated; 95% CI 2,918–3,202; 1,872 observed). We observed a greater decrease in notifications among refugees/migrants compared with non-refugees/migrants (64%, 95% CI 60–67 vs. 39%, 95% CI 36–42), and individuals aged <15 years compared with those aged ≥15 years (60%, 95% CI 57–64 vs. 38%, 95% CI 36–41). We also observed a decrease in the proportion of individuals receiving DST for several drugs.

**CONCLUSIONS:**

These findings underscore the challenges to TB prevention and care during disruption and may be generalizable to the current wartime situation, especially considering the substantial increase in refugees within and leaving Ukraine.

## RÉSUMÉ

**CONTEXTE:**

Nous avons évalué l'impact de la pandémie de COVID-19 sur les notifications de TB en Ukraine, stratifiées en plusieurs sous-groupes.

**CONCEPTION/MÉTHODES:**

Nous avons analysé les données du Programme national de lutte contre la TB de l'Ukraine de janvier 2015 à décembre 2020 à l'aide de modèles de séries chronologiques interrompues. Nous avons comparé les cas observés aux cas contrefactuels estimés si la pandémie n'avait pas eu lieu et les tendances estimées jusqu'en décembre 2020 à l'échelle nationale et selon divers groupes démographiques. Nous avons comparé les proportions de personnes ayant subi un test de sensibilité aux médicaments (DST) en février 2020 et avril 2020 pour évaluer l'impact de la pandémie sur les tests de résistance aux médicaments.

**RÉSULTATS:**

En avril 2020, il y avait 39% (IC à 95% 36–42) de notifications de TB de moins que le contrefactuel estimé (3 060 estimés ; IC à 95% 2 918–3 202 ; 1 872 observés). Nous avons observé une plus grande diminution des notifications chez les réfugiés/migrants par rapport aux non-réfugiés/migrants (64%, IC à 95% 60–67 contre 39%, IC à 95% 36–42), et les personnes âgées de <15 ans par rapport à celles âgées de ≥15 ans (60% ; IC à 95% 57–64 contre 38% ; IC à 95% 36–41). Nous avons également observé une diminution de la proportion de personnes recevant le DST pour plusieurs médicaments.

**CONCLUSIONS:**

Ces résultats soulignent les défis de la prévention et des soins de la TB pendant les perturbations et peuvent être généralisés à la situation actuelle en temps de guerre, en particulier compte tenu de l'augmentation substantielle du nombre de réfugiés à l'intérieur et à l'extérieur de l'Ukraine.

The WHO estimated 1.5 million deaths from TB in 2020, marking the first increase from the previous year in over a decade.^1^ This increase has been attributed to the COVID-19 pandemic and the substantial drop in global TB notifications.^1–4^ A survey of TB and HIV professionals in low- and middle-income countries reported that it has been harder for TB patients and healthcare providers to reach healthcare facilities since the start of the pandemic, with the most common reasons being fear of getting infected with SARS-CoV-2 and transportation and/or movement restrictions.^5^ Thus, this decrease in notifications may not correspond to a drop in actual TB prevalence but rather is an indication that people are not accessing TB care and treatment.

Ukraine is among the 30 high-burden countries for rifampicin-resistant TB (RR-TB) and has one of the highest proportions of TB cases with drug resistance.^6^ It is important to understand the impact of the COVID-19 pandemic on TB notification rates as the ongoing Russo-Ukrainian war causes further disruption to TB care and prevention.^7^ We aimed to quantify the impact of the pandemic on TB notifications in Ukraine, stratified by various demographics including age, sex, refugee/migrant status, drug resistance profiles, and HIV status, using the Ukrainian National TB Program’s (NTP’s) data from January 2015 to December 2020. There is currently no shortage of TB tests and treatment available in Ukraine, yet individuals are still accessing TB care at lower rates (personal communication MD). Thus, if we can identify specific types of people who saw the greatest decrease in notifications during the COVID-19 pandemic, active case finding among these groups could be increased to close the detection gap caused by the war.

## METHODS

### Setting

In 2015, Ukraine’s population was 45,154,036.^8^ The estimated TB incidence was 91/100,000 population in 2015 and 74/100,000 in 2020.^9^ An estimated 31% of new and 45% of previously treated TB cases were RR-TB.^6^

Ukraine announced the first confirmed COVID-19 case on March 3, 2020 and began a quarantine enforced over the entire territory on March 12, 2020. Stricter measures were implemented in April 2020 as cases continued to rise but were lifted in May 2020. Cases began to surge again in August 2020 and continued to increase throughout the year.^10^

### Data sources

Ukraine’s NTP requires clinicians to enter standardized data for each person diagnosed with TB into ‘eTB manager’, an online case registry. Data collected included demographics, HIV status, anatomic site(s) of TB disease, and TB test results. NTP staff downloaded de-identified data on all TB notifications from January 1, 2015, to December 31, 2020 from the eTB Manager in early 2021. Per NTP guidelines, TB disease evaluation includes smear microscopy; mycobacterial culture; Xpert MTB/RIF (Cepheid, Sunnyvale, CA, USA); drug susceptibility testing (DST) to rifampicin, isoniazid, ethambutol, pyrazinamide, and streptomycin for culture-positive isolates; HIV testing; and posterior–anterior and lateral chest radiographs (CXRs).^11^ For a person with RR-TB, a full range of DST for the second-line drugs was available. All RR specimens were tested for fluoroquinolone, injectable drugs, ethionamide, prothionamide, and linezolid. DST for bedaquiline and delamanid was not available at that time. We used World Bank population estimates for yearly country-wide and age- and sex-stratified population numbers (https://data.worldbank.org/country/UA).

### Definitions

We grouped individuals into four age categories: <15, 15–34, 35–54, and ≥55 years. We considered four different drug resistance categories, per WHO pre-October 2020 guidelines: rifampicin-susceptible TB (RS-TB); RR-TB without fluoroquinolone or second-line injectable resistance; isoniazid mono-resistance (H-mono); and pre-extensively drug resistant or extensively drug-resistant TB (pre-XDR or XDR-TB).^12^ Individuals were asked to self-report if they were internally displaced persons (IDPs), migrants, or refugees. We grouped all IDPs, migrants, and refugees together as ‘refugee/migrants’ for analyses because of the small sample sizes in each group. Individuals were defined as a person living with HIV (PLHIV) if their HIV status was confirmed by three rapid tests using different test kits. We considered January 2015–February 2020 to be pre-COVID-19, March 2020 to be a transition period, and April 2020 to be the start of the COVID-19 pandemic. We defined a TB episode as a single occurrence of TB disease, recognizing that people can be reinfected with TB and that one person could contribute more than one episode to our data.

### Statistical analysis

We excluded notifications from March 2020 from the analysis because it was considered a transition period. We calculated the total number of TB notifications per month from January 2015 through December 2020 as well as counts stratified by the following demographic traits: sex, HIV status, refugee/migrant status, employment status, housing status, and age group. HIV, refugee/migrant, employment, and housing status were encoded as affirmative or missing, and missing variables were assumed to be negative.

We modeled the monthly counts of TB episodes using a negative binomial model with population offset and terms for month (Month_i_; continuous number of months since start of the study period), number of months since COVID-19 began (Months since COVID_i_), and an indicator for if COVID-19 was occurring in said month (COVID_i_).^13,14^ The model was expressed as follows:

log(TB notifications_i_) = β_0_ + β_1_(Month_i_) + β_2_(Months since COVID_i_) + β_3_(COVID_i_) + log(Population_i_)

where Population_i_ was the total population in year *i* for all models except age and sex, in which yearly age- and sex-specific populations were used. We estimated the rate ratios and the counterfactual number of TB episodes that would have occurred had COVID-19 not occurred. We calculated standard errors for the counterfactual via the delta method using R package ‘*msm*’ v1.7 (R Computing, Vienna, Austria).^15^ The delta method uses a first-order Taylor approximation to expand a differentiable function of a random variable about its mean to calculate the variance of the said function of the random variable.

An interrupted time series analysis relies on a linear trend before the “interruption” to be able to estimate what would have happened afterwards in its absence. Due to the switch from Xpert^®^ MTB/RIF (Cepheid, Sunnyvale, CA, USA) to Xpert^®^ Ultra (Cepheid) in 2019 (personal communication MD), there was a sudden drop in the use of GeneXpert as well as knock-on effects, leading to a decrease in other DST at the same time (Supplementary Figures S1 and S2). Given the absence of a linear trend, an interrupted time series analysis was not appropriate. Therefore, we calculated the numbers of people diagnosed with TB who received DST and assessed significant differences between February 2020 and April 2020 using χ^2^ tests, recognizing that this cruder method does not estimate a counterfactual for April 2020. All analyses were conducted using R v4.1.2 (R Statistical Foundation).

### Ethics

This study was considered non-human subjects research after review by the Boston University Medical Center Institutional Review Board (IRB), Boston, MA, USA (approval number H-34553) because this study uses routinely collected, de-identified data.

## RESULTS

### Population characteristics

A total of 226,322 TB episodes occurred during the study period. Of these, 161,952 (72.5%) were among males, 50,604 (22.6%) were among PLHIV, 2,397 (1.1%) were among refugees or migrants, 104,901 (46.8%) were among employed people, and 7,967 (3.6%) were among unhoused people. Individuals aged 35–54 years contributed the majority (51.1%) of recorded TB episodes. RS-TB was the most recorded drug resistance category (43.6%), followed by RR-TB (21.8%). A total of 27,568 (12.2%) episodes had an unknown drug resistance status (Table 1). We excluded 3,147 (1.4%) episodes from the following analyses because they occurred during March 2020 (the transition month).

**Table 1.** Characteristics of people diagnosed with TB in Ukraine, January 2015–December 2020.

	*n* (%)
Total, *n*	226,322
Sex	
Female	62,290 (28)
Male	164,032 (72)
HIV status	
Negative	175,129 (77)
Positive	51,193 (23)
Refugee or migrant?	
No	223,900 (99)
Yes	2,422 (1)
Drug resistance status	
RS-TB	98,722 (44)
RR-TB	49,315 (22)
INH-monoresistant	12,695 (6)
RS-TB with second-line resistance	248 ()
Polyresistant	816 ()
Pre-XDR or XDR-TB	37,231 (16)
Unknown	27,568 (12)
Employment status	
Employed	120,421 (53)
Unemployed	105,901 (47)
Housing status	
Housed	218,223 (96)
Unhoused	8,099 (4)
Age group, years	
<15	4,203 (2)
15–34	62,313 (28)
35–54	115,574 (51)
≥55	44,232 (20)

RS-TB = rifampin-susceptible TB; RR-TB = rifampin-resistant TB; INH = isoniazid; XDR-TB = extensively drug-resistant TB.

### National results

**Figure 1.**
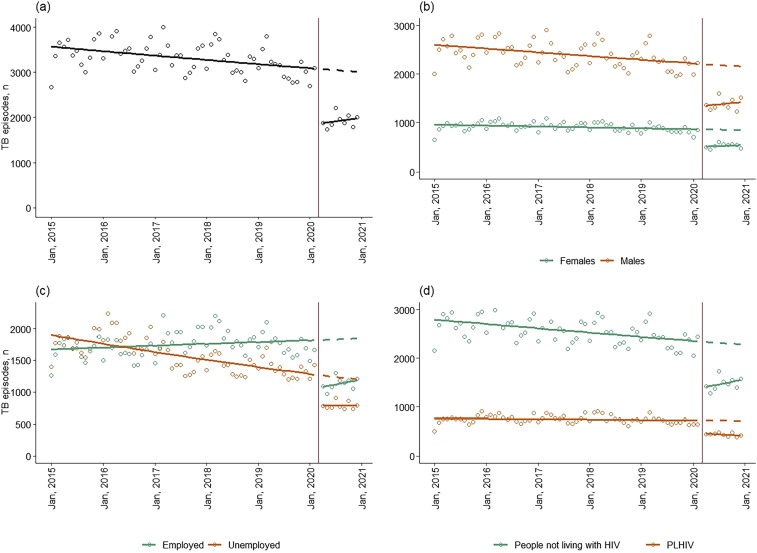
Number of monthly TB notifications in Ukraine, January 2015–December 2020 (dots) and a fitted linear trend lines through these notifications (solid line) separated into pre-COVID-19-pandemic and during the COVID-19 pandemic **A)** nationally, and by **B)** sex, **C)** employment status, and **D)** HIV status. The vertical red line falls on March 2020 to indicate the start of the pandemic in Ukraine and the separation of these two fitted trend lines. The dashed line indicates the predicted monthly notifications if the pandemic had not occurred and notifications had continued the trend observed, January 2015–February 2020.

Before March 2020, TB notifications decreased at a rate of 2.04 cases per 1,000 population per month (95% confidence interval [CI] 0.79–3.29) (Figure 1). In April 2020, 1,872 TB notifications were recorded. This is 38.8% (95% CI 36.0–41.7) lower than expected had pre-pandemic trends held (Table 2). TB notifications continue to increase throughout 2020. In December 2020, 1,995 TB notifications were recorded, compared to 3,010 (95% CI 2,843–3,177) expected notifications, (33.7%, 95% CI 30.0–37.4 fewer than expected). (Table 2).

**Table 2.** Observed and counterfactual estimated TB notifications (in the absence of the COVID-19 pandemic) in April and December 2020 in Ukraine, and the percentage change between these observed and estimated numbers, stratified by demographics. The counterfactual estimates and rate ratios are obtained from the negative binomial interrupted time series model.

	April 2020				December 2020			
	Observed number of TB cases	Counterfactual estimated number of TB cases *n* (95% CI)	Percentage change % (95%CI)	RR (95% CI)	Observed number of TB cases	Counterfactual estimated number of TB cases *n* (95% CI)	Percentage change % (95%CI)	RR (95% CI)
All	1,872	3,060 (2,918–3,202)	38.8 (36.0–41.7)	0.61 (0.53–0.70)	1,995	3,010 (2,843–3,177)	33.7 (30.0–37.4)	0.66 (0.50–0.86)
Sex								
Female	505	861 (821–901)	41.4 (38.6–44.1)	0.60 (0.52–0.69)	477	852 (804–900)	44.0 (40.9–47.1)	0.65 (0.49–0.86)
Male	1,367	2,199 (2,089–2309)	37.8 (34.7–40.9)	0.61 (0.52–0.71)	1,518	2,159 (2,031–2,287)	29.7 (25.5–33.9)	0.67 (0.50–0.89)
HIV status								
HIV-positive	447	729 (691–767)	38.7 (35.5–41.9)	0.64 (0.54–0.75)	423	726 (680–772)	41.7 (38.1–45.4)	0.57 (0.42–0.78)
HIV-negative	1,425	2,333 (2,222–2,444)	38.9 (36.0–41.8)	0.60 (0.52–0.69)	1,572	2,287 (2,158–2,416)	31.3 (27.4–35.2)	0.69 (0.52–0.91)
Refugee/migrant status								
Refugee or migrant	8	22 (20–24)	63.6 (59.8–67.5)	0.83 (0.56–1.23)	9	20 (17–23)	55.0 (49.3–60.7)	0.55 (0.25–1.24)
Not refugee or migrant	1,864	3,039 (2,897–3,181)	38.7 (35.8–41.5)	0.60 (0.53–0.69)	1,986	2,993 (2,826–3,160)	33.7 (30.0–37.4)	0.66 (0.50–0.87)
Employment status								
Employed	1,089	1,821 (1,724–1,918)	40.2 (37.0–43.4)	0.59 (0.50–0.69)	1,205	1,847 (1,729–1,965)	34.8 (30.6–38.9)	0.65 (0.47–0.88)
Unemployed	783	1,260 (1,189–1,331)	37.9 (34.4–41.4)	0.62 (0.52–0.73)	790	1,200 (1,119–1,281)	34.2 (29.8–38.6)	0.66 (0.48–0.92)
Housing status								
Unhoused	78	132 (118–146)	40.9 (34.5–47.4)	0.52 (0.37–0.73)	66	136 (118–154)	51.5 (45.1–57.8)	0.44 (0.23–0.87)
Housed	1,794	2,929 (2,792–3,066)	38.8 (35.9–41.6)	0.61 (0.53–0.70)	1,929	2,876 (2,716–3,036)	32.9 (29.2–36.7)	0.67 (0.51–1.45)
Age group, years								
<15	25	63 (57–69)	60.3 (56.6–64.1)	0.51 (0.37–0.71)	37	64 (57–71)	42.2 (35.6–48.8)	0.68 (0.36–1.26)
15–34	465	729 (690–768)	36.2 (32.8–39.6)	0.62 (0.52–0.72)	484	692 (648–736)	30.1 (25.6–34.5)	0.71 (0.52–1.61)
35–54	1,004	1,643 (1,563–1,723)	38.9 (35.9–41.9)	0.61 (0.52–0.70)	1,096	1,638 (1,542–1,734)	33.1 (29.2–37.0)	0.65 (0.49–0.87)
>55	378	633 (604–662)	40.3 (33.1–47.5)	0.60 (0.52–0.69)	378	632 (598–666)	40.2 (30.8–49.6)	0.60 (0.46–1.32)

RR = risk ratio; CI = confidence interval.

### Demographic results

**Figure 2.**
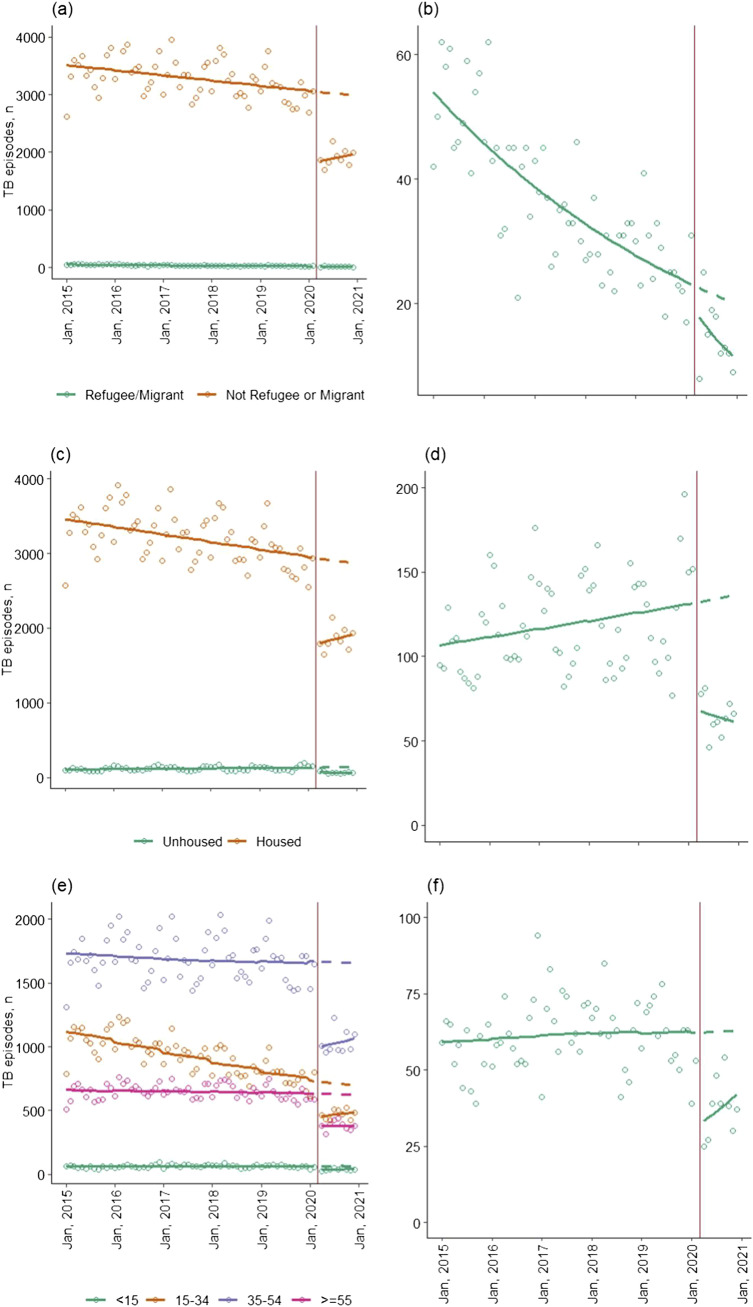
Number of monthly TB notifications in Ukraine, January 2015–December 2020 (dots) and a fitted linear trend lines through these notifications (solid line) separated into pre-COVID-19-pandemic and during the COVID-19 pandemic by **A)** refugee/migrant status, **B)** refugees and migrants only, **C)** housing status, **D)** unhoused individuals only, **E)** age group, and **F)** individuals aged <15 years only. The vertical red line falls on March 2020 to indicate the start of the pandemic in Ukraine and the separation of these two fitted trend lines. The dashed line indicates the predicted monthly notifications if the pandemic had not occurred and notifications had continued the trend observed, January 2015–February 2020.

Prior to March 2020, TB notifications decreased in every subgroup apart from employed individuals (Figures 1 and 2). Fewer TB notifications were observed in April 2020 in every subgroup compared with the expected number in the absence of the pandemic (Figures 1 and 2). Declines were much greater among refugees/migrants (63.6%, 95% CI 59.8–67.5) compared with non-refugees/migrants (38.7%, 95% CI 35.8–41.5), and individuals aged <15 years (59.9%, 95% CI 55.9–63.5) compared with those aged ≥15 years (15–34 years: 35.7%, 95% CI 32.2–39.1; 35–54 years: 39.9%, 95% CI 36.9–42.8; >55 years: 40.1%, 95% CI 32.9–47.3; Table 2). TB notifications were still lower than expected had COVID-19 not occurred in all demographic groups in December 2020. Notably, unhoused individuals saw an even greater percentage decrease in December 2020 (51.5%, 95% CI 45.1–57.8) compared with April 2020 (40.9%, 95% CI 34.5–47.4%; Table 2).

### Drug resistance results

The largest drops in the percentages of people receiving DST were for testing for pyrazinamide and ethambutol resistance (39.6% in February 2020, 22.1% in April 2020, *P* < 0.001; and 53.5% in February 2020, 47.7% in April 2020, *P* < 0.001, respectively) followed by clofazimine resistance (21.9% in February 2020, 16.5% in April 2020, *P* < 0.001), and linezolid (23.0% in February 2020, 17.8% in April 2020, *P* < 0.001; Table 3). There was also a significant decrease in the percentage of individuals receiving DST for isoniazid (54.4% in February 2020, 50.9% in April 2020, *P* = 0.02) and a slight decrease in testing for rifampicin resistance (70.7% in February 2020, 70.0% in April 2020, *P* = 0.60), although this change was not statistically significant. Testing for other drugs appeared to be stable.

**Table 3.** Differences in DST in Ukraine, February 2020 and April 2020.

Drug	Patients who received DST, February 2020 *n*	Proportion of TB episodes, February 2020	Patients who received DST, April 2020 *n*	Proportion of TB episodes, April 2020	Difference in proportions, April 2020 to February 2020	*P*-value for difference
Amikacin	297	0.095	185	0.097	0.00	0.79
Bedaquiline	0	0.000	1	0.001	0.00	0.80
Clofazimine	687	0.219	314	0.165	–0.05	<0.001
Capreomycin	945	0.301	607	0.319	0.02	0.18
Cycloserine	36	0.011	17	0.009	0.00	0.48
Delamanid	3	0.001	1	0.001	0.00	0.99
Ethambutol	1,682	0.535	908	0.477	–0.06	<0.001
Ethionamide	1	0.000	2	0.001	0.00	0.66
Isoniazid	1,708	0.544	968	0.509	–0.03	0.02
Kanamycin	926	0.295	582	0.306	0.01	0.41
Levofloxacin	978	0.311	627	0.330	0.02	0.18
Linezolid	723	0.230	338	0.178	–0.05	<0.001
Moxifloxacin	971	0.309	614	0.323	0.01	0.32
Ofloxacin	282	0.090	158	0.083	–0.01	0.45
Prothionamide	577	0.183	349	0.183	0.00	1
Para-aminosalicylic acid	32	0.010	18	0.009	0.00	0.92
Rifampicin	2,222	0.707	1,331	0.700	–0.01	0.60
Streptomycin	158	0.050	89	0.047	0.00	0.62
Pyrazinamide	1,245	0.396	420	0.221	–0.18	<0.001

DST = drug susceptibility testing.

## DISCUSSION

We observed 39% fewer TB notifications in April 2020 than would have been expected had the COVID-19 pandemic not occurred and TB notifications followed previous trends, with greater decreases among refugees/migrants compared with non-refugees/migrants and individuals aged <15 years compared with those aged 15 years and older. We also observed a significant decrease in the proportion of individuals receiving DST for the first-line drugs ethambutol, isoniazid, and pyrazinamide, as well as decreases for linezolid and clofazimine. Notifications rose slightly throughout 2020 but were still 34% lower in December than expected in the absence of the pandemic. Active case-finding strategies among these groups with larger decreases in observed cases. For example, routine screening for TB among everyone attending refugee centers (regardless of presence of symptoms) and similar surveillance screening in schools, as well as increased DST, may help to increase case detection in these groups of people.

Our findings echo those of other reports that showed decreases in TB notifications at the start of the COVID-19 pandemic.^13,16–24^ There has been debate regarding whether observed changes in notifications can be attributed to reduced case detection versus reduced transmission.^2,25^ Our results for April 2020 cannot have been affected by changes in transmission so quickly and are attributed to decreases in case detection rather than transmission. This decrease in case detection is likely due to a combination of movement restrictions preventing individuals from seeking care for TB symptoms as well as fear of becoming infected with SARS-CoV-2 virus, leading to individuals not wanting to enter healthcare settings.^5^ However, by the end of 2020, it is possible that some of the infection prevention and control interventions to limit COVID-19 transmission (e.g., reducing contacts and mask-wearing) may have also reduced TB transmission.^26^ We did not have information that would allow us to determine what percentage of the drop in notifications in December (compared with what would have been expected in the absence of the pandemic) was attributable to reduced diagnoses versus reduced transmission.

Of particular concern are the decreases observed in the percentages of individuals receiving DST immediately after the start of the COVID-19 pandemic compared with those immediately before. Ukraine has a high burden of RR- and XDR-TB.^27^ Any increase in undiagnosed drug-resistant TB will decrease treatment success rates and potentially increase the transmission of drug-resistant strains. Due to a drop in the use of Xpert tests in mid-2019 (Supplementary Figure S1) caused by the switch to Xpert Ultra in mid-2019 and thus an increase in TB episodes with unknown drug resistance status (Supplementary Figure S2), we failed to assess the impact of the pandemic on drug resistance testing via interrupted time series. Without Xpert, the drug resistance diagnostic pathway requires a series of referrals, thus posing a loss to follow-up risk. However, our results indicate a further drop in the use of DST, as by February 2020, Xpert use had leveled out. Previous global studies have also shown decreases in DR-TB notifications in 2020 compared to 2019,^23,24^ supporting that the decrease we observed in DST, and thus diagnosed DR-TB, was not due to the decrease in use of Xpert.

Ukraine’s NTP faced further disruptions following the COVID-19 pandemic from the Russo-Ukrainian war, beginning in February 2022.^28,29^ We observed a larger decrease in TB notifications among refugees/migrants than among non-refugees/migrants. The number of notifications among refugees/migrants in April 2020 was particularly low, thereby accentuating our results. As such, the percent change and rate ratio in April 2020 suggest somewhat different conclusions. However, despite increasing notifications among migrants and refugees in May 2020, the overall trend post-April 2020 was a more rapid decline during 2020 than before 2020, which is still concerning (Figure 2). It is well documented that undiagnosed TB cases are often disproportionately concentrated in vulnerable populations,^30^ and this group has been made even more vulnerable by the war. Medical organizations report increased difficulties in providing care due to security and transportation issues as well as an influx of almost 6 million internal refugees from Eastern Ukraine.^7^ Although the number of people classed as refugees/migrants in our study was small, if the current situation results in a similar proportional decrease, hundreds, possibly thousands of people with TB will be undiagnosed, leading to devastating impacts on outcomes for those individuals and onward transmission both within Ukraine and elsewhere in Europe. Therefore, increased active case-finding among this population is critical for lowering the TB burden.

A study strength is the use of systematically collected NTP data across a large population with high use of drug susceptibility testing. A limitation of our analysis is that we do not have data on the denominators for every stratified analysis, i.e., we only had access to population data for the entire population of Ukraine and age and sex strata. We, therefore, assumed that the population numbers in these groups did not change over time. Although this may be reasonable for most subgroups, it might not be appropriate for refugee/migrants and employment status. Given that the war in Eastern Ukraine produced a substantial number of IDPs when it began in 2014, the number of IDPs may have reduced over time since then, which we could not incorporate into our analysis. Employment rates also rose slightly in 2018 (https://data.worldbank.org/country/UA); this may have contributed to the positive slope of notification rates seen among employed individuals pre-pandemic. Additionally, our data will be available by the end of 2020. Disruptions due to the war have reduced the resources available for sharing NTP data after 2020. Data from 2021 onwards could provide important insights into how notifications continued to change during the COVID-19 pandemic and how the current war impacted notifications since it began in early 2022. Furthermore, we note that our modeling results cover only the early phase of the COVID-19 pandemic, prior to vaccination availability, which began in February, 2021.^10^

Our data provide a detailed picture of the impact of the COVID-19 pandemic on TB notifications in Ukraine across varying demographic groups. Our finding of particularly large decreases in notifications among migrants and refugees and individuals younger than 15 as well as decreases in DST use highlight the increased susceptibility of these vulnerable people to disruption by war.

## Supplementary Material


