# Supplementary material for: The impact of the COVID-19 pandemic on TB notifications in Ukraine in 2020
Source: IJTLD Open. 2024 Jun 1;1(6):258–65. doi: 10.5588/ijtldopen.24.0194 (PMC11249659; doi:10.5588/ijtldopen.24.0194)

http://dx.doi.org/10.5588/ijtldopen.24.0194

SUPPLEMENTARY DATA

The impact of the COVID-19 pandemic on TB notifications in Ukraine in 2020

Anne N Shapiro[1+], Mariia Dolynska[2], Silvia S Chiang[3, 4], Natasha Rybak[5], Vasyl Petrenko[6], C. Robert Horsburgh Jr[7], Julia Kobe[1], Iana Terleieva[8], Olga Sakalska[8], Helen E. Jenkins[1]

Affiliations:

[1] Department of Biostatistics, Boston University School of Public Health, Boston, USA

[2] NGO Infection Control Ukraine, Kyiv, Ukraine

[3] Department of Pediatrics, Warren Alpert Medical School of Brown University, Providence, Rhode Island, USA

[4] Center for International Health Research, Rhode Island Hospital, Providence, Rhode Island, USA.

[5] Department of Medicine, Warren Alpert Medical School of Brown University, Providence, Rhode Island, USA.

[6] Bogomolets National Medical University, Kyiv, Ukraine

[7] Department of Global Health, Boston University School of Public Health, Boston, USA

[8] Public Health Center of the Ministry of Health, Kyiv City, Ukraine.

[+]Corresponding author. Email: anshap@bu.edu.

Figure S1. Number of TB episodes diagnosed by Xpert, smear or culture, or other test type in Ukraine January 2015-December 2020. The vertical red line falls on March 2020 to indicate the start of the pandemic in Ukraine.

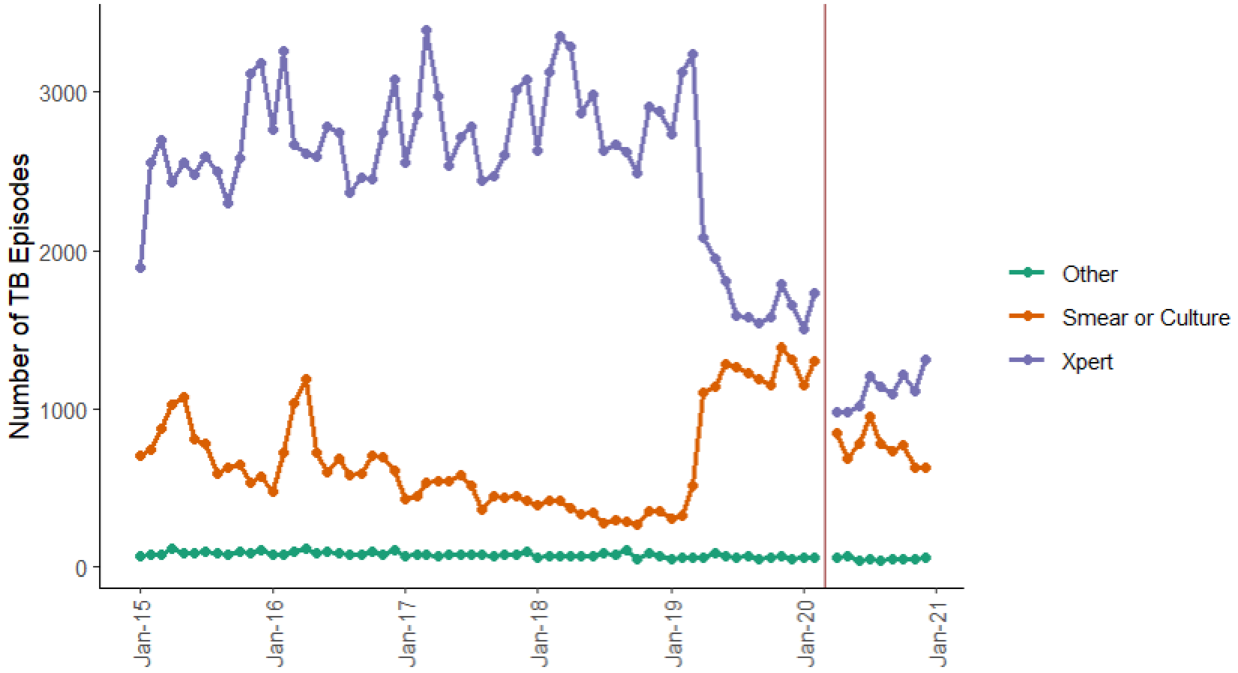

Figure S2. Number of TB episodes by drug resistance status in Ukraine January 2015- December 2020. The vertical red line falls on March 2020 to indicate the start of the pandemic in Ukraine. Drug resistance categories are grouped per WHO pre-October 2020 guidelines: rifampicin-susceptible TB (RS-TB); rifampicin resistant TB without fluroquinolone or second line injectable resistance (RR-TB without FLQ or SLI); isoniazid mono-resistance (H-mono); pre-extensively drug resistant or extensively drug resistant TB (pre XDR or XDR-TB).

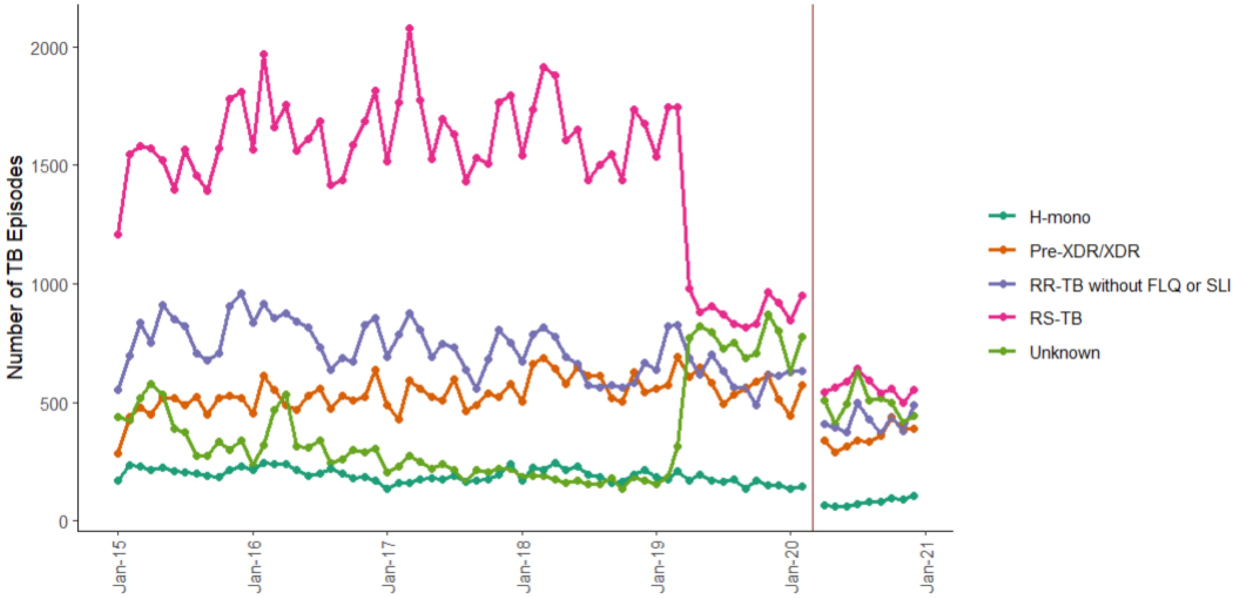

Supplement: Supplementary file 1 [file 0194_supp_data.pdf]
